# Blunt Liver Trauma at Sunnybrook Medical Centre: A 13 Year Experience

**DOI:** 10.1155/1991/86814

**Published:** 1991

**Authors:** Sherif S. Hanna, G. Pagliarello, G. Taylor, H. Miller, H. M. C. Scarth, F. Brenneman

**Affiliations:** Division of General Surgery, Department of Surgery Sunnybrook Medical Centre, H171, 2075 Bayview Avenue University of Toronto Ontario Toronto M4N 3M5 Canada

## Abstract

Between June 1, 1976 and June 30, 1989 The Regional Trauma Unit at Sunnybrook Medical Centre in Toronto, Ontario, Canada received 3730 patients. Of these 335 (9%) sustained a liver injury, 95% being due to blunt trauma. Open peritoneal lavage was performed on 80% of liver trauma patients (267/335), 99% being true positive.

A laparotomy was performed on 97% of patients (324/335). Major surgical treatment was required in 132 patients (41%) and minor treatment in 192 patients (59%). The remaining 11 patients were treated conservatively (n = 3) or died during resuscitation (n = 8).

Morbidity directly related to the liver injury was seen in 29 of 249 surviving patients (11%) although overall morbidity was 27% (67/249). Reoperation was required in 6% (14/249) with abscess or hematoma accounting for 11 of 14 operations.

The overall mortality rate was 26% (86/335). Eighty two percent of patients (n = 276) had a grade I, II or III liver trauma according to Moore’s classification with a mortality of 12% (n = 32). The remaining 18% of patients (n = 59) had a grade IV or V liver trauma with a mortality of 44% (n = 26). Of the 86 deaths, head injury accounted for 48 (56% of deaths); liver hemorrhage for 17 (20%), liver sepsis for (1%) and other causes for 20 deaths (23%). Thus death due to the liver injury itself (hemorrhage and sepsis) occurred in 18 out of 335 patients (5% overall). Head injury accounted for the death of 48 out of 335 patients (14% overall).

Over the past 13 years a trend has occurred at our institution whereby we are seeing less liver trauma in our population of multiply injured patients from 12% (1976–1983) down to 7% (1985–1989); with a gradual decline in overall mortality from 32% (1976–1983) to 19% (1985–1989), whereas the precentage of deaths due to head injuries and liver injury have increased.


*HPB Surgery*, 1991. Vol. 4, pp 49-58
Reprints available directly from the publisher
Photocopying permitted by license only

© 1991 Harwood Academic Publishers GmbH
Printed in the United Kingdom

### BLUNT LIVER TRAUMA AT SUNNYBROOK MEDICAL CENTRE: A 13 YEAR EXPERIENCE

SHERIF S. HANNA,* G. PAGLIARELLO, G. TAYLOR, H. MILLER, H.M.C. SCARTH and F. BRENNEMAN

*Division of General Surgery, Department of Surgery, Sunnybrook Medical Centre, University of Toronto, Toronto, Ontario, Canada*

*(Received 29 May 1990, in final form 24 October 1990)*

Between June 1, 1976 and June 30, 1989 The Regional Trauma Unit at Sunnybrook Medical Centre in Toronto, Ontario, Canada received 3730 patients. Of these 335 (9%) sustained a liver injury, 95% being due to blunt trauma. Open peritoneal lavage was performed on 80% of liver trauma patients (267/335), 99% being true positive.

A laparotomy was performed on 97% of patients (324/335). Major surgical treatment was required in 132 patients (41%) and minor treatment in 192 patients (59%). The remaining 11 patients were treated conservatively (n = 3) or died during resuscitation (n = 8).

Morbidity directly related to the liver injury was seen in 29 of 249 surviving patients (11%) although overall morbidity was 27% (67/249). Reoperation was required in 6% (14/249) with abscess or hematoma accounting for 11 of 14 operations.

The overall mortality rate was 26% (86/335). Eighty two percent of patients (n = 276) had a grade I, II or III liver trauma according to Moore's classification with a mortality of 12% (n = 32). The remaining 18% of patients (n = 59) had a grade IV or V liver trauma with a mortality of 44% (n = 26). Of the 86 deaths, head injury accounted for 48 (56% of deaths); liver hemorrhage for 17 (20%), liver sepsis for 1 (1%) and other causes for 20 deaths (23%). Thus death due to the liver injury itself (hemorrhage and sepsis) occurred in 18 out of 335 patients (5% overall). Head injury accounted for the death of 48 out of 335 patients (14% overall).

Over the past 13 years a trend has occurred at our institution whereby we are seeing less liver trauma in our population of multiply injured patients from 12% (1976–1983) down to 7% (1985–1989); with a gradual decline in overall mortality from 32% (1976–1983) to 19% (1985–1989), whereas the precentage of deaths due to head injuries and liver injury have increased.

KEY WORDS: Hepatic trauma, liver trauma, blunt liver trauma, abdominal trauma

### INTRODUCTION

Liver injury constitutes a major problem in patients suffering from abdominal trauma. The liver is the second most commonly injured organ following blunt abdominal trauma[1]. The most common cause in Canada is blunt injury from motor vehicle accidents where the patients are usually multiply injured[2]. The hemodynamic effect of a major liver injury is profound and taxes the reserves of personnel and blood banks in institutions ranging in size from small hospitals to large regional trauma units. Although minor liver injuries can be simply handled, major injuries continue to cause mortality.

*Address: Dr. S. S. Hanna, Sunnybrook Medical Centre, H171, 2075 Bayview Avenue, Toronto, Ontario, Canada, M4N 3M5

The purpose of this paper was to review the outcome of liver injuries at Sunnybrook Medical Centre (SMC) which serves as a Regional Trauma Unit (RTU) for a large area in southern Ontario, Canada over the past 13 years since the inception of the RTU and to study the epidemiology and changes in morbidity and mortality over this time period. Two previous papers have reported our earlier experience with liver trauma at SMC[3,4] up to June 30, 1985. The present paper updates our entire experience up to June 30, 1989.

### MATERIALS AND METHODS

This is a retrospective review of all patients admitted to the Sunnybrook Medical Centre-Regional Trauma (SMC-RTU) Unit between June 1, 1976 and June 30, 1989. The charts of all patients with liver injury were examined. Data regarding age, sex, mechanism of injury, degree of liver injury, method of treatment, outcome and mechanism of death were all extracted. Peritoneal lavage was performed using an open technique. An infra-umbilical cutdown was performed (supra-umbilical if pelvic fracture was present) with special attention to good hemostasis, and under direct vision a catheter was inserted into the peritoneal cavity. One liter of saline was instilled and then allowed to return by gravity. The lavage fluid was then sent for RBC count, WBC count and amylase level.

Over the 13 years covered by this review the interpretation criteria for lavage positivity with regards to the RBC count have changed. From 1976 - 1983 RBC lavage count >10,000/mm3 constituted a positive lavage. From 1983 -- 1985 a lavage RBC count >20,000/mm3 was considered positive[5]. From 1985 - 1987 peritoneal lavage was considered positive with a lavage RBC count >50,000 cells/mm3. Since July 1987 we do not operate unless the lavage RBC count is >100,000 cells/mm3 which is the standard criterion. Other criteria for positivity of lavage — white blood cell count (>500 cells/mm3) and amylase (>175 u/L) — have remained unchanged throughout this period (1976–1989).

The diagnostic technique in use during that time period was open peritoneal lavage since excellent results have been reported from this institution[5]. The cell count criteria for a positive test have changed during that time period with the RBC count criterion becoming more rigid. Currently an RBC count of >100,000 cells is considered positive.

The liver injury was graded as shown in Table 1, as described by Moore *et al.*[6]. Drainage, hemostatic agents, combined drainage and hemostatic agents or packing were regarded as minor surgical treatment. Repairs requiring of suture, suture and drainage, or resectional debridement were considered as major surgical treatment (Table 2).

### RESULTS

The study period encompassed 13 years during which time 3730 patients were treated at the SMC-RTU. Liver injury was diagnosed in 335 (9%). The liver injury patients had a mean age of 30 and a mean Injury Severity Score of 37. The male:female ratio was approximately 2:1.

**Table 1** Grading System for Liver Trauma, Moore F A *et al.* Reference 6.

| | |
|---|---|
| Class I | Capsular tear<br>Parenchymal fracture <1 cm deep |
| Class II | Parenchymal fracture 1–3 cm deep<br>Subcapsular hematoma <10 cm<br>Peripheral penetrating wound |
| Class III | Parenchymal fracture >3 cm deep<br>Subcapsular hematoma >10 cm<br>Central penetrating wound |
| Class IV | Lobar tissue damage<br>Massive central hematoma |
| Class V | Extensive bilobar disruption<br>Retrohepatic vena cava tear |

**Table 2** Type of surgical treatment in 324 patients who underwent laparotomy

| | *Number of Patients* | *Percentage* |
|---|---|---|
| Minor Surgical Treatment (n = 192) | | 59% |
| No treatment | 48 | 15% |
| Drainage only | 40 | 12% |
| Hemostatic agents only | 43 | 13% |
| Hemostatic agents & drainage | 57 | 18% |
| Packing (± suture) | 4 | 1% |
| Major Surgical treatment (n = 132) | | 41% |
| Suture | 24 | 8% |
| Suture and drainage | 79 | 24% |
| Resectional debridement | 29 | 9 |
| Total | 324 | 100% |

The etiology responsible for the liver injury was blunt trauma in 318/335 (95%) and penetrating trauma in the remaining 17. Of the blunt trauma group, 90% were the result of motor vehicle accidents.

Laparotomy was performed in 324/355 (97%) of patients with liver injury. The remaining 11 patients were treated conservatively (n = 3) or died during resuscitation (n = 8) (Figure 1). Forty-five patients were taken to the operating room directly because of shock, obvious peritonitis, penetrating wounds or ruptured diaphragm. The remaining 267/335 (80%) underwent open peritoneal lavage to diagnose abdominal injury. The true positive rate in this group was 99%.

The liver injuries were classified according to the scheme suggested by Moore[6]. Two hundred and seventy six patients (82%) had a grade I, II or III liver trauma with a mortality of 12% (n = 32). The remaining 59 patients (18%) had a grade IV or V liver trauma with a mortality of 44% (n = 26).

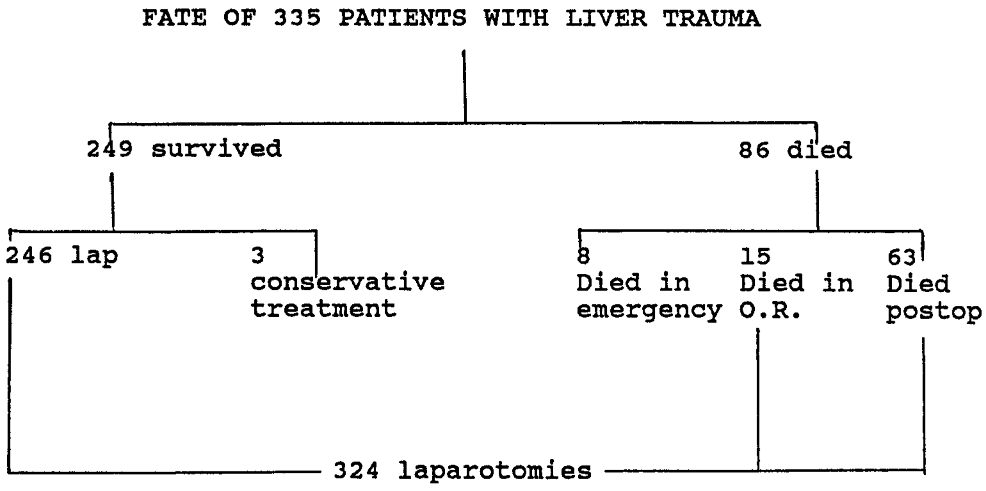

**Figure 1** Fate of 335 patients with liver trauma.

The liver injuries were managed by several modalities. Minor surgical treatment was required in 192/325 (59%). Major surgical treatment was used in 132/324 (41%). The specific treatments used are listed in Table 2.

The overall mortality in the entire group of patients with liver injuries was 86/335 (26%). Eight of these died during resuscitation in the emergency department (one of these died from liver hemorrhage, 2 from head injuries and the rest from other causes). Seventy eight patients died in hospital and the causes of death are listed in Table 3. The majority of deaths were due to head injury. Liver related deaths included 17 due to liver hemorrhage and 1 due to sepsis for a liver related death rate of 18/335 (5.4%). The overall death rate was 12% in the minor injury group (class I, II or III) and 44% in the major liver injury group (classes IV and V).

A total of 83 complications were encountered in 67 of 249 surviving patients (27%). The commonest complications were sepsis and bile fistula or collection. The specific complications are listed in Table 4.

**Table 3** Cause of Death in 78 Patients Who Died After Admission

| | *Number of Patients* |
|---|---|
| Head Injury | 46 |
| Liver Hemorrhage | 16 |
| Liver Sepsis | 1 |
| Shock (non-hepatic bleeding) | 4 |
| Respiratory failure | 3 |
| Retroperitoneal Hematoma | 3 |
| Myocardial Contusion | 2 |
| Ruptured Aorta | 2 |
| Pulmonary Embolism | 1 |
| | 78 |

**Table 4** Complications seen in 67 of the 249 Surviving Patients

| | *Number of Episodes* |
|---|---|
| Sepsis | 21 |
| Biliary Fistula/Bile collection | 10 |
| Disseminated Intravascular Coagulation | 8 |
| Liver Failure | 7 |
| Perihepatic Hematoma | 5 |
| Renal Failure | 5 |
| Pulmonary Embolus | 4 |
| Other | 23 |
| Total Number of Complications | 83 |

For the purpose of comparison, the study period was arbitrarily divided into 3 times periods. The number of cases, the associated laparotomy rates, death rates, etc. are listed in Table 5. The proportion of patients with liver injury has decreased from 12% to 9%. The liver related mortality rate has increased from 15% to 23%.

### *Discussion*

The diagnosis of liver trauma, in our experience, is usually made at a laparotomy for a positive minilap. Eighty per cent of our liver trauma patients underwent a minilap before laparotomy. Our true positive rate was 99% with a 1% false negative rate. We rely heavily on the minilap since physical examination is unreliable in the multiply injured patient especially in the presence of head injury, alcohol or drugs. Over the years, we have become more rigid in our interpretation of lavage RBC results. Currently we only operate if the lavage RBC count is >100,000/cells mm3. Our experience with CT scans in the diagnosis of blunt abdominal trauma is gradually evolving[7] but we continue to rely on open peritoneal lavage as the gold standard for diagnosis.

Recently Little *et al.*[8,9] have estimated that 25–30% of adults sustaining blunt liver trauma can be managed without surgery. This requires good organ imaging, close monitoring in an intensive care unit and the availability of surgical intervention at short notice to deal with the complications. Out of 335 patients in this series, only 3 patients were managed non-operatively. All 3 had small subcapsular or intrahepatic hematomas that were diagnosed late and managed without surgery with a successful outcome in all. We do not advocate a non-operative approach to liver trauma in polytraumatized patients, such as is seen in our series, because of the high incidence of associated injuries both inside and outside the abdomen. On the other hand, in cases of isolated blunt abdominal trauma in hemodynamically stable patients without peritonitis, careful observation may be employed if liver imaging reveals a minor or moderate injury. In our entire experience of liver and other abdominal injuries, we have not had a single mortality due to a negative laparotomy.

A problem that has plagued the literature pertaining to liver injury is the lack of a uniform classification system for liver injuries. This makes comparison of published results difficult, if not impossible. It is therefore hoped that in the future literature on liver trauma will refer to the patient's liver injury in a standard fashion. We have

**Table 5** Comparison of liver trauma series at Sunnybrook Medical Centre over three time periods from 1976–1989

| Series | Reference number | Time Period M/D/Year | Total number of trauma patients | No. of patients with liver trauma | % of patients with liver trauma | % of patients with blunt liver trauma | % of patients having open peritoneal lavage (number) | % of patients under going laparotomy (number) | % of patients requiring minor surgical treatment (number) | % of patients requiring major surgical treatment (number) | % of patients with morbidity (number) | % of episodes of morbidity (number) | % mortality number | % of deaths due to head injury (number) | % of deaths due to liver hemorrhage (number) |
|---|---|---|---|---|---|---|---|---|---|---|---|---|---|---|---|
| One | 3 | 6/1/76 — 3/31/83 | 1173 | 145 | 12% | 97% | 113/145 78% | 134/145 92% | 79/134 59% | 55/134 41% | 29/98 30% | 45/98 46% | 47/145 32% | 24/47 51% | 7/47 15% |
| Two | 4 | 4/1/83 — 6/30-85 | 843 | 75 | 9% | 96% | 62/75 83% | 75/75 100% | 50/75 67% | 25/75 33% | 13/58 22% | 13/58 22% | 17/75 23% | 9/17 53% | 5/17 29% |
| Three | * | 7/1/85 — 6/30/89 | 1714 | 115 | 7% | 94% | 92/115 80% | 115/115 100% | 63/115 55% | 52/115 45% | 25/93 27% | 25/93 27% | 22/115 19% | 15/22 68% | 5/22 23% |
| Total | * | 6/1/76 — 6/30/89 | 3730 | 335 | 9% | 95% | 267/335 80% | 324/335 97% | 192/324 59% | 132/324 41% | 67/249 27% | 83/249 33% | 86/335 26% | 48/86 56% | 17/86 20% |

* Represents this paper found Moore's classification of liver injury to be useful and simple[6] and recommend its adoption (Table 1). When we divided our cases according to his system, we saw a gradual rise in mortality. In our experience patients with grade I, II or III liver trauma had an overall mortality of 12%, versus a mortality of 44% for grade IV or V liver trauma. Very recently Moore proposed a modification of his original system which is more detailed and has been proposed by the organ injury scaling committee of the American Association for the Surgery of Trauma[10]. We have not had any experience with the latter system because of its very recent introduction.

Hemorrhage is the commonest cause of death after major liver trauma, accounting for 82% of deaths in Beal's series[11]. How many of these deaths due to hemorrhage are preventable? This is a very difficult question to answer from the literature or from personal experience. In this series of 335 patients, 86 patients died, of whom 17 died due to liver hemorrhage. Three of these 17 deaths (18% of deaths due to liver hemorrhage) were thought to be potentially preventable. One had a moderate injury that was potentially salvageable and the other patient's injury was under-estimated at the original laparotomy resulting in a delayed fatal hemorrhage. A third patient had delayed transport to hospital and died just after arrival to hospital and conceivably could have survived if transported more rapidly. The remaining deaths due to liver hemorrhage were thought to be non-preventable either due to the severity of the injury itself ($n = 5$), other serious injuries ($n = 8$) or underlying cirrhosis ($n = 1$).

The vast majority (80–90%) of liver injuries can be handled simply using sutures and drains. The remaining 10–20% of injuries are severe and require expert management. There is general agreement on the basic principles of management of hepatic trauma[2,3,4]. Aggressive resuscitation, rapid surgical control of bleeding, debridement of devitalized liver tissue, adequate external drainage using sump or suction drains, aggressive treatment of associated injuries, correction of coagulopathy and expert supportive postoperative care are all required. Deep lacerations should be opened in order to directly control bleeding vessels in the depths of the wound.

Formal hepatic lobectomy in liver trauma carries a mortality of 50%[11]. This is because patients requiring such treatment are actively bleeding and are often in hemorrhagic shock, usually with other associated injuries and often suffer from coagulopathy due to massive blood transfusions. In view of this, we do not advocate this approach unless the injury makes it mandatory for anatomic reasons. We advocate a more conservative, non-resectional approach to severe liver trauma whenever possible. The Pringle maneuver[12] of clamping the hepatoduodenal ligament can be performed with impunity for up to an hour in normothermic, non-cirrhotic patients and even longer in hypothermic patients[13]. This allows the surgeon to repair the liver in a relatively avascular field. If liver tissue is already devitalized or avulsed partially or completely, a resectional debridement can be performed by completing the laceration and removing the dead portion of liver. We have recently been successful in treating a severe case of liver trauma by opening of the laceration and applying the hemostatic agent tissue fibrin glue. Formerly such a case would most likely have been treated by major resectional debridement.

Despite the above areas of general agreement there are still areas of controversy relating to the management of liver injuries. These include the use of packing, the role of hepatic artery ligation and hepatic vein and retrohepatic caval injuries.

Figure 2 shows an algorithm for the operative management of severe hepatic bleeding.

Packing of liver injuries is very useful in small or remote hospitals with minimal blood bank facilities. It is also useful in large hospitals in the presence of severe coagulopathy[14,15]. Laparotomy pads or Kling bandage is packed above and below the liver but not into the laceration. Packs should be removed at surgery 48–72 hours later when the coagulation abnormalities have been corrected[14], although one author has left the packing for 7–10 days apparently without any increase in the incidence of infection[15]. Others have not found packing useful[16]. We have used packing recently for 48–72 hours in 4 patients with liver trauma and coagulopathy and have found it extremely useful in this setting.

Hepatic artery ligation was enthusiastically adopted by many surgeons with little experience in liver trauma after it was initially advocated[17]. It will only control deep arterial bleeding which is a rare occurrence in liver trauma. Most liver hemorrhage comes from the venous side. In this entire series of 335 cases, hepatic artery ligation was not used even once. In general, we do not advocate its use.

Trauma to the main hepatic veins or retrohepatic cava remains the most difficult, complex and controversial problem in the management of liver trauma and carries a mortality of 40 – 100%. Such an injury is suspected if the Pringle maneuver is unsuccessful in controlling hepatic bleeding and/or if the injury extends into the bare area of the liver. Hardy[18] examined 50 victims of fatal blunt liver trauma in Melbourne, Australia and discovered that it is uncommon for major hepatic veins

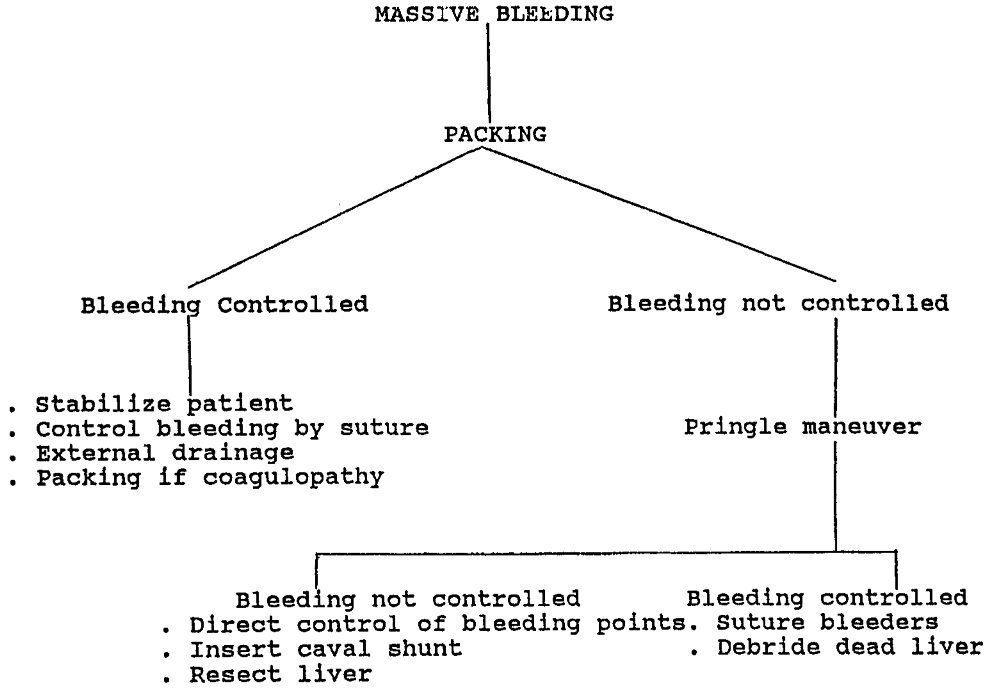

**Figure 2** Algorithm for the intra-operative control of massive hepatic bleeding.

to be damaged; but when the right hepatic vein is damaged it usually tears at the junction of its intra and extra hepatic course, 1–2 cm from its termination due to a shearing force. He also discovered that the middle hepatic vein is usually damaged within the liver due to antero-posterior compression of the spine. We have recently seen an example of this in a patient who fell nine stories from an apartment building. In our experience, it is also uncommon for major hepatic vein injury to occur secondary to blunt liver trauma. An anterior tear of the main right hepatic vein may be directly repaired after mobilizing the liver by dividing its ligamentous attachments. Major hepatic venous injury occurring within the substance of the liver or retrohepatic caval injuries can be managed either by vascular isolation of the liver as originally described by Schrock and Blaisdell in 1968[19]; or by direct repair after finger fracture and resection of the liver. Despite using the Schrock shunt or its various modifications, the mortality of this injury remains very high. In this series of 335 patients vascular isolation was used on 6 patients with one survivor (83% mortality).

Buechter *et al.*[20] have recently reported on 20 patients with retrohepatic caval injuries (11 penetrating and 9 blunt traumas). Overall mortality was 75%. A shunt was used in 10 patients with a mortality of 90% and in 10 non-shunted patients, the mortality was 60%. They conclude that direct exposure and repair of retrohepatic caval injuries carries a lower mortality and suggest that rapid application of the direct exposure technique would provide better survival than shunt techniques. Beal[11] also recently reported on 121 patients with severe, mostly blunt liver trauma with an overall mortality of 32%. Eighty two per cent of deaths were due to exsanguination. Liver packing was used on 35 patients of whom 20 had hepatic vein or retrohepatic caval injuries and 5 had severe bilobar parenchymal disruptions; with a mortality of 15%. In 10 patients shunts were used with a 60% mortality. In view of the above two reports and our own disappointing results with caval shunting in retrohepatic caval injuries, we recommend that, if such an injury is encountered, packing be initially tried. If successful, the patient is closed and the packs removed in 24–48 hours. If packing is unsuccessful then the Pringle maneuver is used and direct exposure and repair of the injury is recommended at the time of initial laparotomy.

In summary most liver injuries can be simply managed with hemostatic agents, sutures and drainage. Resectional debridement is indicated for peripheral injuries with partial or complete avulsion of devascularized liver tissue. Packing is useful in those cases associated with coagulopathy. The major remaining unresolved management issue in blunt liver trauma is how to best manage hepatic vein and retrohepatic caval injuries. Packing should be tried first. If it fails to control the bleeding, the injury should be exposed and repaired. Caval shunting is associated with a high mortality.

### *References*

1. Elerding, S.T. and Moore, E.E. (1980) Recent experience with trauma of the liver. *Surg. Gynaecol. Obstet.* **150**, 853–855
2. Hanna, S.S. and Jirsch, D.W. (1977) Management of Hepatic Injury. *Can. Med. Assoc.J.* **117**, 352–353
3. Hanna, S.S., Maheshwari, Y. and Harrison, A.W. *et al.* (1985) Blunt Liver Trauma at the Sunnybrook Regional Trauma Unit. *Can. J. Surg.* **28**, 22–223

4. Hanna, S.S., Gorman, P. and Harrison, A.W. *et al* (1987) Blunt Liver Trauma at Sunnybrook Medical Centre. *Journal of Trauma*, **27**, 965–969
5. McLellan, B.A., Hanna, S.S. and Montoya, D.R. *et al* (1985) Analysis of Peritoneal Lavage Parameters in Blunt Abdominal Trauma. *J. of Trauma*. **25**, 393–399
6. Moore, F.A., Moore, E.E. and Seagraves, A. (1985) Non-resectional Management of Major Hepatic Trauma: An evolving concept. *Am. J. Surg*. **150**, 725–729
7. Pagliarello, G., Hanna, S.S. and Gregory, W. *et al* (1987) Abdomino-pelvic Computerized Tomography and Open Peritoneal Lavage in Patients with Blunt Abdominal Trauma: A Prospective Study. *Can.J.of Surg*. **30**, 10–12
8. Little, J.M., Fernandes, A. and Tait, N. (1986) Liver Trauma. *Aust. NZ. J. Surg*. **56**, 613–20
9. Little, J.M. (1988) Trauma to the liver, pancreas and porta hepatis: Aggressive and Conservative Management. In Progress in Surgery of the Liver, Pancreas and Biliary System. Edited by S. Bengmark. Martinus NIJHOFF Publishers. Dordrecht, Boston, Lancaster; Chapter 7, pp 129–141
10. Moore, E.E., Schackford, S.R. and Pachter, H.L. *et al.* (1989) Organ Injury Scaling: Spleen, Liver and Kidney. *J. of Trauma*, **29**, 1664–1666
11. Beal, S.L. (1990) Fatal Hepatic Hemorrhage: An Unresolved Problem in the Management of Complex Liver Injuries. *J. of Trauma*, **30**, 163–169
12. Pringle, J.H. (1908) Notes on the arrest of hepatic hemorrhage due to trauma. *Ann. Surg*. **48**, 541–9
13. Huguet, C., Nordlinger, B. and Block, P. *et al.* (1978) Tolerance of the human liver to prolonged normothermic ischemia: A biological study of 20 patients submitted to extensive hepatectomy. *Arch. Surg*. **113**, 1448-1451
14. Feliciano, D.V., Mattox, K.C. and Burch, J.M. *et al.* (1986) Packing for Control of Hepatic Hemorrhage. *J. of Trauma*, **26**, 738–743
15. Baracco-Gandolfo, V., Vidarte, O. and Baracco-Miller, V. *et al.* (1986) Prolonged Closed Liver Packing in Severe Hepatic Trauma: Experience with 36 patients. *J. of Trauma*, **26**, 754–756
16. Ivatury, R.R., Nallathambi, M. and Gunduz, Y. *et al.* (1986) Liver Packing for Uncontrolled Hemorrhage: A Reappraisal. *J. of Traume*, **26**, 744–753
17. Mays, E.T. (1976) The Hazards of suturing certain wounds of the liver. *Surg. Gynaecol. Obstet.* **143**, 201–4
18. Hardy, K.J. (1972) Patterns of Liver Injury after Fatal Blunt Trauma. *Surg. Gynaecol. Obstet*. **134**, 39–43
19. Schrock, T., Blaisdell, F.W. and Mathewson, Jr. C. (1968) Management of Blunt Trauma to the Liver and Hepatic Veins. *Arch. Surg*. **96**, 698–704
20. Buechter, K.J., Sereda, D. and Gomez, G. *et al.* (1989) Retrohepatic Vein Injuries: Experience with 20 cases. *J. of Trauma*, **29**, 1969–1704

*(Accepted by S Bengmark 27 October 1990)*

